# Nomogram Incorporating Preoperative Testing Markers for the prediction of Early Recurrence for Colorectal Liver Metastases with Neoadjuvant Chemotherapy followed by Hepatectomy

**DOI:** 10.7150/jca.65677

**Published:** 2022-03-14

**Authors:** Qichen Chen, Yizhou Zhang, Xingchen Li, Zhen Huang, Hong Zhao, Jianqiang Cai

**Affiliations:** 1 Department of Hepatobiliary Surgery, National Cancer Center/National Clinical Research Center for Cancer/Cancer Hospital, Chinese Academy of Medical Sciences and Peking Union Medical College, Beijing, 100021, China.; 2 Department of Thoracic Surgery, National Cancer Center/National Clinical Research Center for Cancer/Cancer Hospital, Chinese Academy of Medical Sciences and Peking Union Medical College, Beijing, 100021, China.

**Keywords:** colorectal liver metastasis, recurrence, neoadjuvant chemotherapy, nomogram

## Abstract

**Background:** This study aims to develop a nomogram to predict early recurrence in colorectal liver metastases (CRLM) patients who received neoadjuvant chemotherapy (NAC) followed by hepatectomy.

**Methods:** The primary cohort included 144 CRLM patients treated in the same ward, and 40 CRLM patients from another ward were allocated to the validation cohort. The minimum *p* value method was used to find the optimal cut-off point for early recurrence. Predictors of early recurrence were determined by univariate analysis and multivariate logistic regression. Nomogram for early recurrence was constructed and area under the receiver operating characteristic curve (AUROC), calibration curves, and decision curve analysis (DCA) were used to evaluate its performance.

**Results:** The optimal cut-off point for the definition of early recurrence was 11 months. Patients with early recurrence have significantly worse post-recurrence survival (median 23.5 months vs. 35.7 months, *P*=0.012) and overall survival (median 31.8 months vs. 59.0 months, *P*<0.001). Preoperative creatinine ≥ 63.5 µmol/L (*P*=0.035), synchronous liver metastasis (*P*=0.030), non-R0 resection (*P*=0.044), postoperative complications (*P*=0.009) and NAC Cycles ≥ 4 (*P*=0.003) were independent predictors of early recurrence. A nomogram with favorable discrimination (AUROC of 0.754 in the primary cohort and 0.882 in the validation cohort) and calibration was constructed to predict early recurrence. The DCA results also indicated good clinical applicability.

**Conclusions:** This is the first study to identify elevated preoperative creatinine as an independent predictor of early recurrence of CRLM treated with hepatectomy after NAC. This study developed a nomogram that particularly incorporated preoperative creatinine to predict early recurrence in CRLM patients receiving NAC to aid clinical decision-making.

## Introduction

Surgical resection is the only treatment that offers potential cure for colorectal liver metastases (CRLM) 1. A Five-year survival rate of 40-56% can be reached with complete surgical resection 2-6. However, more than one-half of patients who received hepatectomy for CRLM would develop recurrence, and most recurrences were detected within 2 years after surgery 7-9. Several studies reported a significant negative impact of early recurrence on the survival of CRLM patients. However, various definitions of early recurrence were used in these studies, with the cut-off value of recurrence-free survival (RFS) ranging from 6 months to 2 years 10-14. Statistically determined cut-off values of early recurrence have been explored to better demonstrate its impact on survival. *Imai et al*. determined 8 months as the optimal cut-off value of early recurrence by the minimum *p* value approach 7. *Liu et al.* reported 12 months as the optimal cut-off point for CRLM patients with preoperative chemotherapy 15.

Neoadjuvant chemotherapy (NAC) followed by surgery and adjuvant chemotherapy was an option for initially resectable CRLM 2. NAC is believed to prolong survival in a subset of patients with a high risk of disease recurrence 16, and improve PFS 2. NAC may also help prevent patients with disease progression under systematic therapy from receiving futile invasive local therapy 17. Therefore, the use of NAC on initially resectable CRLM is prevalent.

As early recurrence of CRLM after hepatectomy is associated with significantly worse survival, prediction of early recurrence is crucial. Previous studies identified multiple predictive factors of early recurrence, including patient demographics (e.g. age 7), clinicopathological characteristics (e.g. bilobular distribution 10, number of metastases 7, 11, 12), features of intervention (e.g. surgical margin 10, 12), and cancer-specific biomarkers [e.g. carcinoembryonic antigen (CEA) 10, carbohydrate antigen 19-9 (CA 19-9) 7. However, none of the previous studies incorporated preoperative blood markers to predict early recurrence. Preoperative blood markers [e.g. gamma-glutamyl transpeptidase (GGT), D-dimer] were easily obtained from routine blood tests and recently found to be novel predictors of the prognosis of CRLM patients 18, 19. We hypothesized that changes in preoperative blood marker levels could also be predictive of early recurrence. Nomograms are reliable and convenient tools of predicting hazards and facilitate clinical decision-making in CRLM management. Several nomograms were already established to predict the prognosis of CRLM patients with curative surgery after NAC 15, 20-22. However, these established nomograms focused on predicting postoperative complications or survival, and nomograms focusing on the prediction of early recurrence are lacking. Constructing a nomogram specifically incorporating preoperative blood markers to predict early recurrence may aid clinicians to better distinguish patients of high risk and plan for more personalized postoperative treatment.

This study aims to construct a nomogram incorporating preoperative blood markers to predict early recurrence in CRLM patients with neoadjuvant chemotherapy followed by hepatectomy.

## Methods

### Study population

This study was approved by our institutional review board and informed consents were obtained from all participants involved in this study. We retrospectively examined the medical records of 513 CRLM patients who received hepatectomy from December 2008 to September 2020, at Cancer Hospital, Chinese Academy of Medical Sciences. The inclusion criteria of this study were: (1) histologically confirmed colorectal adenocarcinoma liver metastasis; (2) received hepatectomy after NAC; (3) developed recurrence. The exclusion criteria of this study were: (1) received neoadjuvant radiotherapy; (2) loss to follow-up; (3) with missing clinical data. Demographics, clinicopathologic features, treatment characteristics, and follow-up information were collected from medical records. Peripheral venous punctures within one week before surgery were used to analyze preoperative blood markers, including GGT, D-dimer, ALT, AST, ALB, FIB, LDH, and creatinine. These preoperative blood markers were included in this study as they were routinely tested and were considered to have a potential association with the prognosis of CRLM patients. This set of preoperative blood markers included in this study was determined before data collection. Synchronous liver metastasis is defined as liver metastasis detected before or at diagnosis of the primary tumor, or during surgery for the primary tumor.

### Chemotherapy and Surgical Treatment

As described previously 22, NAC was recommended to patients with one or more factors that are considered to increase the risk of recurrence, including primary lymph node metastasis at diagnosis, synchronous presentation of the primary tumor and liver metastases, and more than one liver lesion. The chemotherapy regimens were FOLFOX (5-flurouracil, leucovorin, and oxaliplatin), FOLFIRI (5-flurouracil, leucovorin, and irinotecan), or XELOX (capecitabine and oxaliplatin), with or without bevacizumab or cetuximab. NAC toxicities included symptoms of the blood system, the digastric tract, or the nervous system. Second-line chemotherapy was given to patients with inadequate control of the disease or severe toxicity during the first-line chemotherapy. Patients who received oxaliplatin in either first-line or second-line chemotherapy were considered as receiving oxaliplatin-based regimen. After the NAC regimens were completed, liver surgeries were performed in four to six weeks. Pathological response to NAC was defined as tumor regression grade (TRG) 1-3 after hepatectomy 23. Surgical resection of three or more liver segments was defined as major liver resection.

### Outcomes and follow-up

Postoperative complications included wound dehiscence, infection (incision site, pulmonary, urinary infection, etc.), anastomotic fistula, hemorrhage, bile leak, ascites, liver failure, urine retention, ileus, atrial fibrillation, and hydrothorax. Postoperative complications were recorded until hospital discharge.

As described previously, patients were followed up at regular intervals after surgery 22. The first follow-up was done 1 month after hepatectomy. The following follow-ups were done at intervals of 3 months within the first 2 years, 6 months between the second to the fifth year, and once per year thereafter. Recurrence or disease progression was scanned by monitoring level of CEA and imaging. OS and PFS were defined from the date of hepatectomy to death or detection of disease progression, respectively, or to the last available follow-up. Post-recurrence survival (PRS) was calculated from the detection of recurrence to death or to the last available follow-up.

### Statistical analysis

The Mann-Whitney U test was used to compare continuous variables, and the Chi-square test or the Fisher's exact test was used to compare categorical factors when considered appropriate. The minimum *p* value method based on subsequent PRS was used to find the optimal cut-off value to define early recurrence of CRLM. In brief, the study population was categorized into potential early recurrence cohort and potential late recurrence cohort by various cut-off values of RFS with regular intervals. PRS was compared for each evaluated cut-off value by the log-rank test. The cut-off value of RFS with minimum *p* value in the log-rank test was considered as the optimal cut-off point to define early recurrence. OS and PFS were analyzed by the Kaplan-Meier method and compared by the log-rank test. When converting continuous variables into categorical factors, the cut-off values were determined by the ROC curve, or by clinical relevance. Potential predictors determined by the univariate analysis with *P* < 0.10 were examined in the multivariate analysis. Multivariate logistic regression was performed to identify independent predictors of early recurrence. Statistical significance was determined as two-tailed *P* < 0.05. Independent predictors identified in the multivariate analysis were used to construct the nomogram. In brief, the independent predictor with the highest beta coefficient in the logistic regression model was assigned 100 points in the nomogram, and the scores of other independent predictors were assigned as the beta coefficient of the predictor divided by the highest beta coefficient among the predictors 24. The possibilities of developing early recurrence with various total nomogram scores were calculated from the logistic regression model and plotted on the bottom of the nomogram. In both the primary cohort and the validation cohort, the area under the receiver operating characteristic curve (AUROC) was calculated to evaluate the discrimination of the nomogram. Calibration curves and the Hosmer-Lemeshow test was used to evaluate calibration of the nomogram, and decision curve analysis (DCA) was performed to evaluate the clinical applicability of the nomogram. All statistical analyses were performed by SPSS software version 22 (Armonk NV, USA) and R software (http://www.r-project.org).

## Results

### Clinicopathological characteristics of patients with recurrence

The primary cohort consists of 144 patients from ward 1 of the Department of Hepatobiliary Surgery in the final study population. Patients in the primary cohort received either staged resection or simultaneous resection. In the case of staged resection, hepatectomy is only performed after complete surgical removal of the primary tumor. One hundred forty-four patients were included in the primary cohort. The median age was 54.5 (IQR: 49.3-61.8) years old in the primary cohort. Sixty-two (43.1%) patients had comorbidities. Seventeen patients (11.8%) had primary tumors in the right hemicolon. One hundred and ten patients (76.4%) had primary lymph node metastases at diagnosis, and 81 patients (56.3%) had bilobular distribution of liver lesions. The patients in the primary cohort had 3 (IQR: 2-5) liver metastases at median, and the median of the largest diameter of liver lesions was 2.8 (IQR: 1.9-4.0) cm. Major liver resection was performed for 122 (84.7%) patients. The median duration of the surgery was 346.5 (IQR: 262.5-423.8) min, and the median volume of intraoperative blood loss was 300 (IQR: 150-500) ml. In this cohort, 21 participants also exhibited extrahepatic metastases.

In the preoperative blood testing, the median level of CEA was 8.1 (IQR: 3.4-36.1) ng/ml in the primary cohort. The median preoperative GGT value was 38.0 (IQR: 24.2-63.8) U/L. The median preoperative D-dimer value was 0.48 (IQR:0.28-0.81) mg/L. The median level of preoperative alanine aminotransferase (ALT) was 20.5 (IQR: 15.0-30.0) U/L. The median level of preoperative aspartate aminotransferase (AST) was 24.0 (IQR: 18.0-29.8) U/L. The median level of preoperative albumin (ALB) was 41.8 (IQR: 38.9-44.2) g/L. The median level of preoperative creatinine was 63.0 (IQR: 56.0-72.8) μmol/L. Oxaliplatin-based NAC regimens were given to 94 (65.3%) patients, and 54 patients (37.5%) received bevacizumab or cetuximab. The median number of cycles of NAC was 5 (IQR: 4-7). NAC toxicities were recorded in 127 patients (88.2%). Fifty-four patients (34.7%) exhibited a favorable pathological response to NAC.

**Figure 1:**
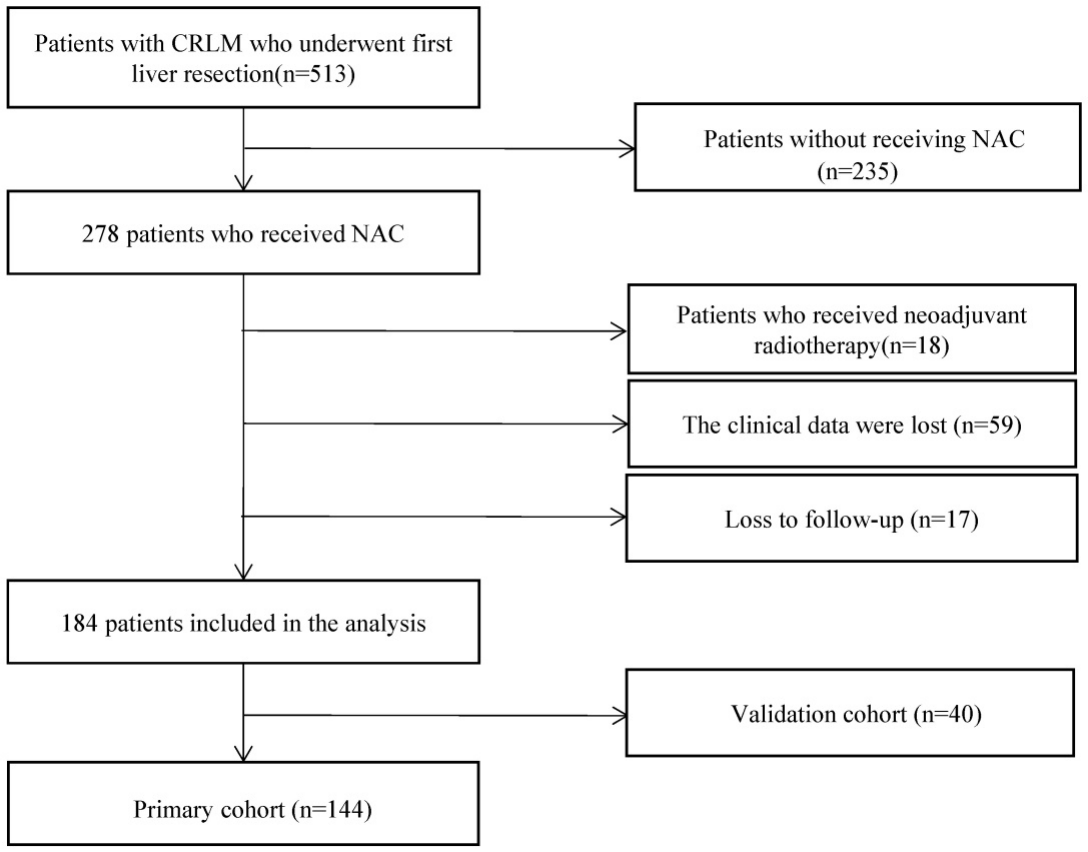
Flowchart of the study population

We included 40 patients from ward 2 of the Department of Hepatobiliary Surgery in this study as the validation cohort. All patients in the validation cohort received simultaneous resection from January 2018 to September 2020. The flowchart of the study population was shown in **Figure 1**. Details of demographics and clinicopathological characteristics of the primary cohort and the validation cohort are shown in **Table 1**.

### Defining Early and Late Recurrence

**Figure 2:**
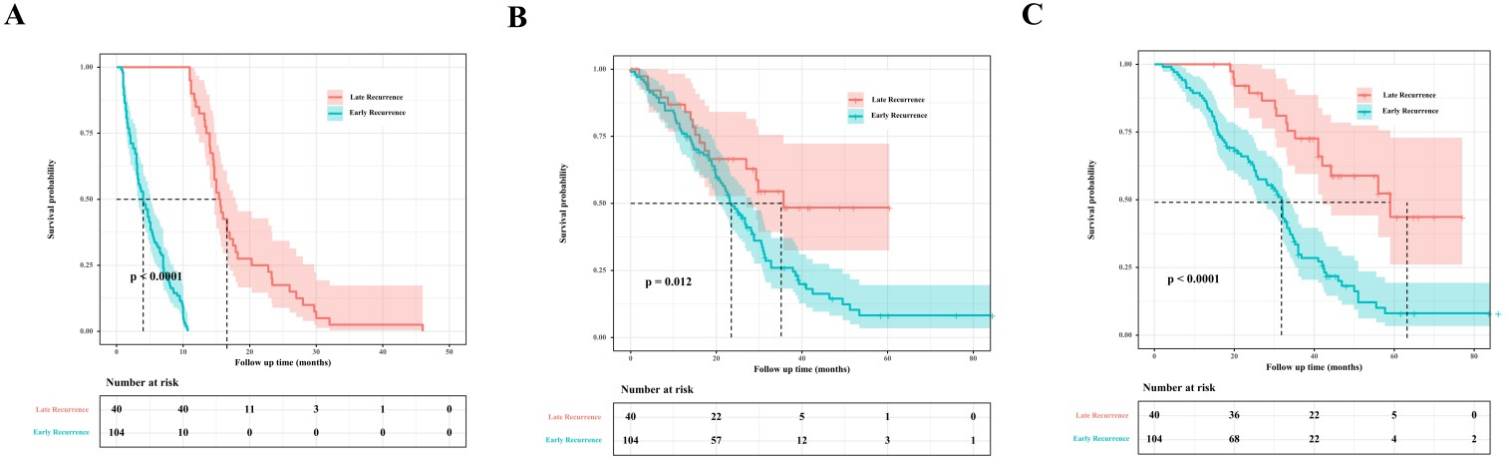
Survival analysis of early recurrence vs. late recurrence. **A.** RFS analysis. **B.** PRS analysis. **C.** OS analysis.

The cut-off thresholds examined for the definition of early recurrence and corresponding RFS, PRS, and OS are shown in **Table 2**. Based on subsequent PRS, the optimal cut-off threshold of RFS to define early recurrence was 11 months (*P*=0.012). For patients with early recurrence (<11mo, n=104, 72.2%), the median RFS was 4.0 months (95% CI 2.9-5.1), and the median PRS was 23.5 months (95% CI 19.9-27.1). Patients with late (≥11mo) recurrence (n=40, 27.8%) had a median RFS of 15.4 months (95% CI 14.2-16.0), and a median PRS of 35.7 months (95% CI 9.4-12.2). The median OS of patients with early recurrence was 31.8 months (95% CI 28.7-34.9), and the median OS of patients with late recurrence was 9.0 months (95% CI 37.3-80.8). Patients with early recurrence had significantly unfavorable RFS (*P <* 0.0001, **Figure 2A**), PRS (*P*=0.012, **Figure 2B**), and OS (*P <* 0.0001, **Figure 2C**). When compared to patients with late recurrence, more patients with early recurrence had elevated preoperative creatinine (*P*=0.047), primary tumor in the right hemicolon (*P*=0.032), synchronous liver metastasis (*P*=0.024), more number of liver metastases (*P*=0.004), non-R0 resection (*P*=0.041), postoperative complications (*P*=0.008) and more NAC cycles (*P*=0.001).

### Factors Associated with Early Recurrence

The best cut-off value for preoperative creatinine was 63.5 μmol/L with a sensitivity of 54.8% and specificity of 65.0%. Fifty-seven of 104 patients (54.8%) with preoperative creatinine values exceeding 63.5 μmol/L developed early recurrence, while 14 of 40 patients (35.0%) with preoperative creatinine values less than 63.5 μmol/L had early recurrence (*P*=0.033). We excluded RAS mutational status from further analyses as this variable was only available in 102 (70.8%) patients of the primary cohort and 29 (72.5%) patients in the validation cohort. The univariate analysis (**Table 3**) identified preoperative creatinine ≥ 63.5 μmol/L (*P*=0.035), synchronous liver metastasis (*P*=0.030), non-R0 resection (*P*=0.044), postoperative complications (*P*=0.009), and NAC Cycles ≥ 4 (*P*=0.003) to be associated with early recurrence. A trend of early recurrence was also observed in patients with the primary tumor in the right hemicolon, with more than one liver metastases, had bilobular distribution of liver metastases, or received radiofrequency ablation (RFA) (*P* < 0.1).

All potential predictors (*P* < 0.1) mentioned above were retained in subsequent multivariate analysis. Preoperative creatinine ≥ 63.5 μmol/L (OR = 2.362, 95% CI: 1.027-5.435, *P* = 0.043), synchronous liver metastasis (OR = 3.416, 95% CI: 1.139-10.251, *P* = 0.028), postoperative complications (OR =3.261, 95% CI: 1.399-7.599, *P* = 0.006), and NAC cycles ≥ 4 (OR =3.858, 95% CI: 1.548-9.612, *P* = 0.004) were independently predictive of early recurrence (**Table 3**).

### Nomogram construction for early recurrence

**Figure 3:**
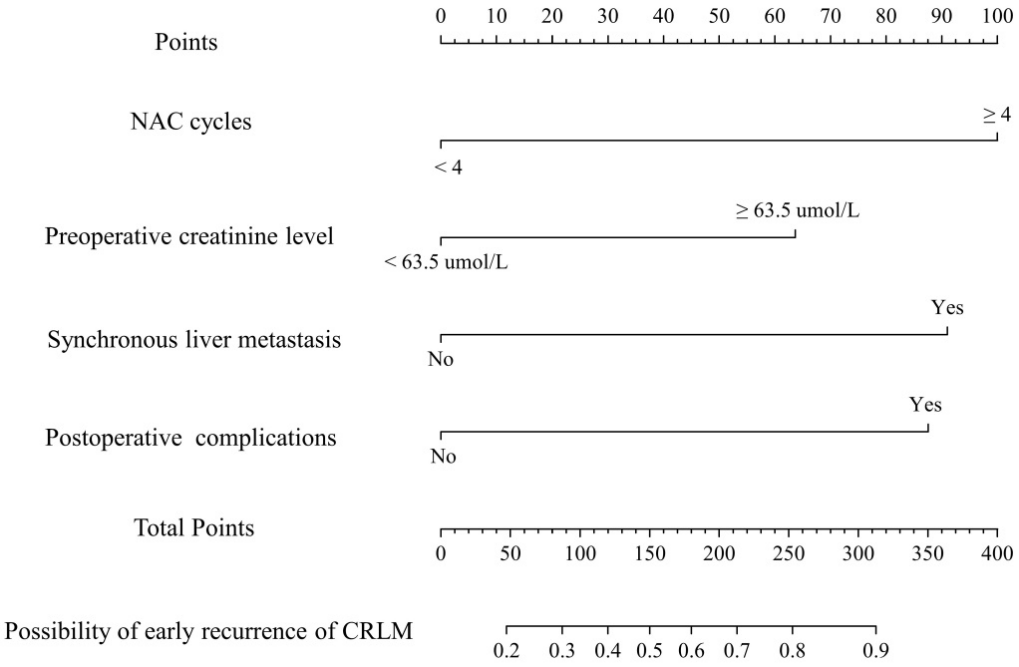
Nomogram predicting the probability of early recurrence in colorectal liver metastases (CRLM) patients.

**Figure 4:**
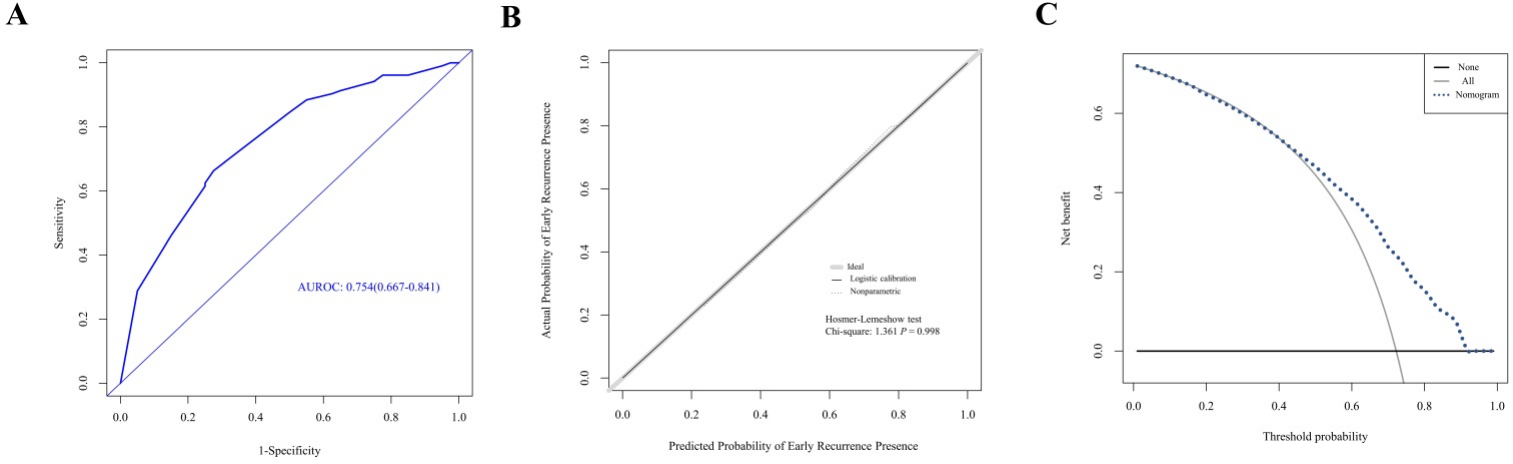
** Evaluation of nomogram in the prediction of early recurrence in the primary cohort. A.** The ROC curves of the nomogram. **B.** The calibration curves for predicting early recurrence presence. **C.** The DCA analysis.

**Figure 5:**
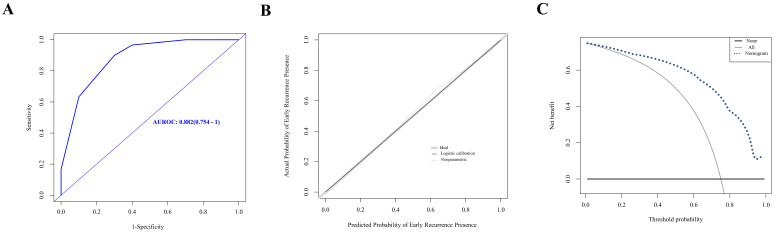
** Evaluation of nomogram in the prediction of early recurrence in the validation cohort. A.** The ROC curves of the nomogram. **B.** The calibration curves for predicting early recurrence presence. **C.** The DCA analysis.

A nomogram was established with the four independent predictors of early recurrence identified by the multivariate analysis **(Figure 3)**. The specific scores were assigned to each independent predictor as follows: preoperative creatinine ≥ 63.5 μmol/L, 64; synchronous liver metastasis, 91; NAC cycles ≥ 4, 100; and postoperative complications, 88. The cut-off threshold of the nomogram score was set to 217 as determined by the ROC curve. In the primary cohort, the sensitivity of the nomogram was 0.663, and the specificity was 0.725. The nomogram showed acceptable ability to predict early recurrence, with an AUROC value of 0.754 (95% CI: 0.667-0.841, **Figure 4A**). Calibration curves and the Hosmer-Lemeshow test showed that the nomogram had favorable calibration (chi-square: 1.36, *P* = 0.998, **Figure 4B**). The DCA result also indicated that nomograms showed good clinical applicability (**Figure 4C**). In the validation cohort, high AUROC (0.882, 95% CI: 0.754 - 1) (**Figure 5A**) was noticed, and good calibration was performed between the predictions by the nomograms and the actual observations (**Figure 5B**). The DCA result in the validation cohort revealed good clinical applicability (**Figure 5C**).

## Discussion

Nomogram plays an important part in guiding personalized treatment. It offers a reliable and convenient prediction of prognosis. Several previous studies have established nomograms for the prediction of prognosis of patients with CRLM and underwent NAC. *Imai et al.* constructed a nomogram to predict OS of patients who presented with initially unresectable liver metastases and received preoperative chemotherapy 20. *Liu et al.* created a nomogram to predict disease-free survival (DFS) 15. The existing nomograms for CRLM mainly focused on the prediction of PFS or OS, while to the best of our knowledge, this is the first study to incorporate preoperative blood markers in the construction of a nomogram focusing on the prediction of early recurrence of CRLM treated with NAC.

In this study, we identified preoperative creatinine ≥ 63.5 μmol/L, synchronous liver metastasis, postoperative complications, and NAC cycles ≥ 4 as four independent predictive factors of early recurrence.

Creatinine is an endogenous biomarker of renal impairment. The levels of serum creatinine can be obtained easily from routine blood tests. The prognostic value of creatinine was confirmed in various malignancies of the urinary system 25, 26, or other systems 27-29. Elevated serum creatinine is associated with an increased risk of recurrence in colorectal cancer patients 30. Even with creatinine level within the normal range, the underlying impaired creatinine clearance can be predictive of unfavorable chemotherapy response and time to progression in metastatic colorectal cancer patients with an unspecified site of metastasis 31. However, evidence of the predictive value of preoperative creatinine on the prognosis of CRLM is lacking. No previous study has reported the prognostic impact of preoperative serum creatinine in patients with CRLM. This is the first study to identify elevated preoperative creatinine as an independent predictor of early recurrence of CRLM treated with hepatectomy after NAC. The correlation between preoperative creatinine and early recurrence can be explained as follows: Elevated creatinine reflects impaired creatinine clearance, which is associated with NAC toxicity. Patients with impaired creatinine clearance who received capecitabine or 5-FU might accumulate their metabolites and may require dose reduction to prevent adverse effects, thereby reducing the efficacy of NAC 32-34. To date, creatinine is rarely reported in studies investigating prognostic factors of CRLM. As preoperative creatinine can be obtained easily from routine blood tests, further studies should be done to examine its potential predictive value on OS or PFS of CRLM patients.

Synchronous CRLM, accounting for approximately one-half of CRLM cases 1, 6, 12, 35-37, is generally considered to be more aggressive, associated with more disseminated disease stage, and have a worse prognosis compared to CRLM patients with metachronous presentation 1, 36-38. The worse tumor biology of synchronous CRLM 39 may alter its pattern of recurrence. A greater recurrence rate in the abdominal cavity was observed in patients with synchronous CRLM 38, while several other studies reported synchronous CRLM to be related to worse DFS 40-42. Hence, it is not surprising that synchronous liver metastasis was identified as an independent predictor of early recurrence, which is in line with a previous study 12.

This study identified postoperative complications as the only surgical factor independently associated with early recurrence. Postoperative complications are known to have a negative impact on the survival and recurrence of CRLM patients 43-45. In the study of *Kaibori et al.*, postoperative complication was the only independent predictor of early recurrence of CRLM 13. Severe postoperative complications may prolong the immunosuppression caused by major surgeries, and delay the beginning of adjuvant chemotherapy, which is considered to facilitate disease recurrence 13. In the present study, the postoperative complication rate reached a relatively high 52.8% (76 in 144), which suggested that better control of postoperative complications may play an important part in the prevention of early recurrence.

NAC cycles ≥ 4 is the only chemotherapy-related factor independently predictive of early recurrence. Patients who received more cycles of NAC might have a less favorable disease stage, poorer response to first-line chemotherapy regimen, and could be involved in more complicated surgeries. Furthermore, prolonged NAC duration may introduce chemotherapy-associated liver injuries 46. Six or more cycles of preoperative oxaliplatin-based chemotherapy were significantly associated with a higher incidence of sinusoidal injury 47. The long-term impact of chemotherapy-associated liver injuries remains unclear, but they were considered to decrease survival indirectly through poor tumor response 48. Of note, the present study did not show a significant correlation between postoperative chemotherapy and early recurrence. The protective effect of adjuvant chemotherapy in CRLM patients treated with hepatectomy after NAC requires further investigation.

As nomogram offers a simple graphical presentation of statistical prediction models, it is favored by both clinicians and patients and proved useful for various types of cancer 24. This nomogram would enable clinicians to conveniently evaluate the risk of early recurrence of individual CRLM patients, and allow personalized adjustment in further treatment planning. Changes in planned adjuvant chemotherapy or monitoring strategy based on the nomogram scores of every single patient can be feasible. Patients with high nomogram scores may require stronger adjuvant chemotherapy and more frequent monitoring. Patients with low nomogram scores may be saved from receiving unnecessary invasive treatment or adjuvant chemotherapy.

This study has several limitations. First, this study is limited by its retrospective design. Causality cannot be inferred confidently in a retrospective observational study due to potential residual confounding. Second, although many factors including preoperative blood markers were incorporated in this study, it is still possible that unmeasured factors would affect early recurrence. Third, the data is collected from a single institution and exposed to potential selection bias. Although the nomogram exhibited acceptable predictability in our internal validation, external validation at other institutions is preferred to strengthen our results. Finally, the cohort may not be of enough size to demonstrate statistical significance for some variables. A prospective study with a larger cohort might resolve these limitations, validate our results and provide stronger evidence.

## Conclusion

This is the first study to identify elevated preoperative creatinine as an independent predictor of early recurrence of CRLM treated with hepatectomy after NAC. This study developed a nomogram that particularly incorporated preoperative creatinine to predict early recurrence in CRLM patients receiving NAC. This nomogram exhibits favorable discrimination and calibration to aid clinical decision-making.

## Figures and Tables

**Table 1:** Demographics, Clinicopathologic, and Treatment Characteristics of all patients with Recurrence

Variety	Primary Cohort				Validation Cohort			
	All patients (n=144)	Early recurrence (n=104)	Late recurrence (n=40)	*P*	All patients (n=40)	Early recurrence (n=30)	Late recurrence (n=10)	*P*
**Demographics and clinicopathological features**								
Age, median (IQR)	54.5 (49.3-61.8)	55.0 (50.0-62.0)	54.0 (48.3-60.8)	0.547	58.5 (52.5-63.0)	56.5 (53.0-61.8)	63.0 (52.3-67.0)	0.222
Male, n (%)	94 (65.3)	70 (67.3)	24 (60.0)	0.409	24 (60.0)	20 (66.7)	4 (40.0)	0.136
BMI≥24kg/m^2^, n (%)	76 (52.8)	59 (56.7)	17 (42.5)	0.125	16 (40.0)	11 (36.7)	5 (50.0)	0.456
Comorbidity, n (%)	62 (43.1)	45 (43.3)	17 (42.5)	0.933	16 (40.0)	13 (43.3)	3 (30.0)	0.456
ASA score 3-4, n (%)	19 (13.2)	16 (15.4)	3 (7.5)	0.210	6 (15.0)	5 (16.7)	1 (10.0)	0.609
Primary site in colon, n (%)	77 (53.5)	55 (52.9)	22 (55.0)	0.820	22 (55.0)	16 (53.3)	6 (60.0)	0.714
Right hemicolon, n (%)	17 (11.8)	16 (15.4)	1 (2.5)	0.032	7 (17.5)	3 (10.0)	4 (40.0)	0.031
Poor differention, n (%)	39 (27.1)	30 (28.8)	9 (22.5)	0.443	16 (40.0)	12 (40.0)	4 (40.0)	1
T3-T4 stage, n (%)	137 (95.1)	99 (95.2)	38 (95.0)	0.962	37 (92.5)	28 (93.3)	9 (90.0)	0.729
Primary lymph node metastasis, n (%)	110 (76.4)	81 (77.9)	29 (72.5)	0.496	32 (80.0)	25 (83.3)	7 (70.0)	0.361
Synchronous liver metastasis, n (%)	126 (87.5)	95 (91.3)	31 (77.5)	0.024	39 (97.5)	29 (96.7)	10 (100.0)	0.559
Diameter of largest liver metastases, median (IQR)	2.8 (1.9-4.0)	3.0 (2.0-4.0)	2.2 (1.6-4.0)	0.244	3.0 (1.8-4.5)	3.5 (2.4-4.89)	1.8 (1.5-2.7)	0.020
Number of liver metastases, median (IQR)	3.0 (2.0-5.0)	4.0 (2.0-5.0)	2.0 (1.0-3.0)	0.004	2.0 (1.0-4.0)	2.5 (2.0-4.0)	2.0 (1.0-3.5)	0.250
Bilobar liver distribution, n (%)	81 (56.3)	63 (60.6)	18 (45.0)	0.091	21 (52.5)	15 (50.0)	6 (60.0)	0.583
Extrahepatic metastases, n (%)	18 (12.5)	12 (11.5)	6 (15.0)	0.574	7 (17.5)	6 (20.0)	1 (10.0)	0.471
KRAS mutation^a^, n (%)	30 (20.83)	24 (23.1)	6 (15.0)	0.086	15 (51.7)	10 (47.6)	5 (62.5)	0.474
**Preoperative laboratory testing levels**								
Preoperative CEA, median (IQR)	8.1 (3.4-36.1)	9.1 (3.4-39.4)	5.7 (3.4-20.8)	0.320	7.3 (3.8-21.2)	7.3 (4.1-19.6)	7.0 (3.2-41.0)	0.975
Preoperative GGT, median (IQR)	38.0 (24.2-63.8)	38.0 (25.0-65.0)	33.5 (22.5-58.5)	0.556	41.0 (29.5-62.0)	45.5 (33.0-64.3)	36.0 (25.5-43.3)	0.206
Preoperative D-dimer, median (IQR)	0.48 (0.28-0.81)	0.50 (0.28-0.83)	0.42 (0.26-0.76)	0.403	0.35 (0.23-0.62)	0.39 (0.22-.61)	0.32 (0.28-0.67)	0.963
Preoperative ALT, median (IQR)	20.5 (15.0-30.0)	20.0 (15.0-28.8)	21.5 (14.3-32.0)	0.595	20.0 (13.0-39.3)	16.0 (12.3-37.3)	29.0 (16.8-57.5)	0.122
Preoperative AST, median (IQR)	24.0 (18.0-29.8)	23.5 (18.0-28.0)	25.0 (17.0-31.8)	0.489	25.0 (19.0-33.3)	24.0 (19.0-30.8)	32.5 (21.8-52.8)	0.067
Preoperative ALB, median (IQR)	41.8 (38.9-44.2)	41.7 (39.0-44.3)	42.0 (36.8-43.9)	0.370	43.3 (40.6-46.2)	43.8 (40.5-46.4)	42.5 (41.8-44.5)	0.606
Preoperative FIB, median (IQR)	3.1 (2.6-3.7)	3.1 (2.6-3.7)	3.2 (2.8-3.7)	0.568	3.1 (2.8-3.7)	3.0 (2.7-3.7)	3.3 (3.0-3.6)	0.399
Preoperative LDH, median (IQR)	177.5 (150.0-213.8)	177.0 (150.0-212.8)	180.0 (152.3-217.8)	0.808	191.0 (161.5-222.0)	180.5 (160.5-219.3)	206.0 (180.0-283.8)	0.349
Preoperative creatinine, median (IQR)	63.0 (56.0-72.8)	65.0 (58.0-74.8)	60.5 (52.3-67.8)	0.047	66.5 (58.3-75.3)	68.0 (56.0-75.5)	61.5 (60.0-73.0)	0.743
**Surgery and chemotherapy details**								
Heterochronous resection, n (%)	40 (27.8)	28 (26.9)	12 (30.0)	0.712	0 (0.0)	0 (0.0)	0 (0.0)	NA
R0 resection, n (%)	85 (59.0)	56 (53.8)	29 (72.5)	0.041	31 (77.5)	24 (80.0)	7 (70.0)	0.512
Major liver resection, n (%)	122 (84.7)	88 (84.6)	34 (85.0)	0.954	29 (72.5)	23 (76.7)	6 (60.0)	0.307
Concomitant RFA, n (%)	33 (22.9)	28 (26.9)	5 (12.5)	0.065	1 (2.5)	1 (3.3)	0 (0.0)	0.559
Operation time (min), median (IQR)	346.5 (262.5-423.8)	349.0 (285.0-431.8)	332.5 (238.0-420.0)	0.377	375.0 (303.8-430.3)	395.0 (320.0-452.0)	328.0 (252.5-358.8)	0.061
Blood loss (ml), median (IQR)	300 (150.0-500.0)	300.0 (200.0-500.0)	200.0 (100.0-400.0)	0.186	200.0 (200.0-525.0)	300.0 (200.0-575.0)	200.0 (125.0-275.0)	0.162
Postoperative complications, n (%)	76 (52.8)	62 (59.6)	14 (35.0)	0.008	28 (70.0)	22 (73.3)	6 (60.0)	0.426
Oxaliplatin based NAC regimen, n (%)	94 (65.3)	64 (61.5)	30 (75.0)	0.129	31 (79.5)	22 (73.3)	9 (100.0)	0.082
NAC cycles, median (IQR)	5.0 (4.0-7.0)	6.0 (4.0-8.0)	4.0 (3.0-6.0)	0.001	6.0 (3.8-6.3)	6.0 (5.3-7.0)	3.0 (2.3-4.5)	0.002
Preoperative targeted therapy, n (%)	54 (37.5)	42 (40.4)	12 (30.0)	0.249	28 (70.0)	24 (80.0)	4 (40.0)	0.017
Second-line NAC, n (%)	28 (19.4)	23 (22.1)	5 (12.5)	0.192	10 (25.0)	6 (20.0)	4 (40.0)	0.206
NAC toxicities, n (%)	127 (88.2)	91 (87.5)	36 (90.0)	0.677	20 (60.6)	15 (57.7)	5 (71.4)	0.509
Pathological response, n (%)	50 (34.7)	35 (33.7)	15 (37.5)	0.664	35 (100.0)	25 (100.0)	10 (100.0)	NA
Postoperative chemotherapy, n (%)	88 (61.1)	62 (59.6)	26 (65.0)	0.553	27 (67.5)	22 (73.3)	5 (50.0)	0.172

a. KRAS status was available in 102 patients of the primary cohort and 29 patients of the validation cohort.

**Table 2:** Evaluated Cut-off Thresholds for defining Early and Late Recurrence based on the Prognosis after Recurrence

Evaluated Cut-off	*P*	Potential Early Recurrence Cohort				Potential Late Recurrence Cohort			
		N	RFS (mo)	PRS (mo)	OS (mo)	N	RFS (mo)	PRS (mo)	OS (mo)
2	0.742	26	1.2	23.2	24.9	118	7.9	26.7	35.2
3	0.179	34	1.4	22	23.6	110	8.6	27	36
4	0.108	49	1.9	22	23.6	95	10	27	36.8
5	0.314	61	2.5	22.2	24.9	83	10.6	27	36.8
6	0.183	69	3.0	22.6	25.4	75	11.2	28	41
7	0.114	74	3.0	22.6	25.5	70	11.7	28.3	41
8	0.186	85	3.2	23.5	30.2	59	14	28.8	42
9	0.083	89	3.3	23.5	30.2	55	14.1	29.8	42.3
10	0.056	94	3.5	24.3	30.7	50	14.6	29.8	51
11	0.012	104	4.0	23.5	31.8	40	15.4	35.7	51.9
12	0.023	110	4.4	24.3	32	34	16	-	59
13	0.035	111	4.4	24.9	32	33	16.6	-	59
14	0.128	114	4.7	25.3	32.1	30	16.6	35.7	59
15	0.686	121	4.9	26	32.1	23	18.2	27	56
16	0.559	126	5.1	26	32.1	18	22.8	27	56

**Table 3:** Univariable and Multivariable Logistic Regression for associations between Risk Factors and Early Recurrence of CRLM after Resection (<11 mo)

Factor	Unitivariate analysis		Multivariate analysis	
	*P*	OR (95%CI)	* P*	OR (95%CI)
**Demographics and clinicopathological features**				
Age ≥60 years	0.756	1.133(0.514-2.499)		
Male	0.410	0.729(0.343-1.548)		
BMI≥24kg/m^2^	0.128	1.774(0.849-3.707)		
Comorbidity	0.933	1.032(0.494-2.157)		
ASA score 3-4	0.220	2.242(0.616-8.159)		
Primary site in colon	0.820	0.918(0.442-1.910)		
Right hemicolon	0.062	7.091(0.908-55.367)	NS	NS
Poor differentiation	0.444	1.396(0.594-3.283)		
T3-T4 stage	0.962	1.042(0.194-5.602)		
Primary lymph node metastasis	0.496	1.336(0.580-3.077)		
Synchronous liver metastasis	0.030	3.065(1.117-8.405)	0.028	3.416(1.139-10.251)
Diameter of largest liver metastases ≥ 3 cm	0.168	1.684(0.803-3.531)		
Multiple liver metastases	0.067	2.128(0.950-4.770)	NS	NS
Bilobar liver distribution	0.094	1.878(0.899-3.923)	NS	NS
Extrahepatic metastases	0.575	0.739(0.257-2.125)		
**Preoperative laboratory testing levels**				
Preoperative CEA ≥10 ng/ml	0.269	1.531(0.719-3.261)		
Preoperative GGT ≥ 38 U/L	0.635	1.194(0.575-2.477)		
Preoperative D-dimer ≥0.48 mg/L	0.224	1.578(0.756-3.295)		
Preoperative ALT ≥ 20.5 U/L	0.710	0.871(0.420-1.807)		
Preoperative AST ≥ 24 U/L	0.420	0.739(0.354-1.542)		
Preoperative ALB ≥ 41.8 g/L	0.710	0.871(0.420-1.807)		
Preoperative FIB ≥ 3.15 g/L	0.710	0.871(0.420-1.807)		
Preoperative LDH ≥ 177.5 U/L	0.710	0.871(0.420-1.807)		
Preoperative creatinine ≥ 63.5 μmol/L	0.035	2.252(1.058-4.796)	0.043	2.362(1.027-5.435)
**Surgery and chemotherapy details**				
Heterochronous resection	0.712	0.860(0.385-1.919)		
R0 resection	0.044	0.443(0.200-0.979)	NS	NS
Major liver resection	0.954	0.971(0.351-2.687)		
Concomitant RFA	0.072	2.579(0.919-7.241)	NS	NS
Operation time ≥ 346.5 min	0.710	1.149(0.554-2.383)		
Blood loss ≥ 300 ml	0.229	1.568(0.753-3.264)		
Postoperative complications	**0.009**	**2.741(1.284-5.854)**	0.006	3.261(1.399-7.599)
Oxaliplatin based NAC regimen	0.132	0.533(0.235-1.208)		
NAC cycles ≥ 4	0.003	3.560(1.534-8.263)	0.004	3.858(1.548-9.612)
Preoperative targeted therapy	0.251	1.581(0.723-3.453)		
Second-line NAC	0.198	1.988(0.699-5.653)		
NAC toxicities	0.678	0.778(0.238-2.544)		
Pathological response	0.664	0.845(0.396-1.805)		
Postoperative chemotherapy	0.553	0.795(0.372-1.697)		

NS, not significant.

## Abbreviations

CRLM: colorectal liver metastasis
NAC: neoadjuvant chemotherapy
AUROC: area under the receiver operating characteristics curve
DCA: decision curve analysis
CEA: carcinoembryonic antigen
CA 19-9: carbohydrate antigen 19-9
GGT: gamma-glutamyl transpeptidase
TRG: tumor regression grade
OS: overall survival
RFS: recurrence-free survival
PRS: post-recurrence survival
ALT: alanine aminotransferase
AST: aspartate aminotransferase
ALB: albumin
DFS: disease-free survival
